# Captopril in Congenital Chloride Diarrhoea: A Case Study

**Published:** 2015-03

**Authors:** Shoeb Bin Islam, Ramendra Nath Mazumder, Mohammod Jobayer Chisti, Sharifuzzaman, Lubaba Sahreen, Tahmeed Ahmed, Nur Haque Alam

**Affiliations:** icddr,b, GPO Box 128, Dhaka 1000, Bangladesh

## Abstract

An 11 months 22 days old girl presented with a history of watery diarrhoea since birth, failure to thrive, and developmental delay. Her diagnosis was congenital chloride diarrhoea (CCD) with raised level of chloride (>90 mmol/L) in stool in the absence of cystic fibrosis. Management of CCD included replacement of NaCl, KCl, and correction of dehydration. Diarrhoea of the patient was resolved with Captopril, which was initially provided to the patient for managing heart failure. To our knowledge, this is the first reported case of CCD that shows the beneficial effect of Captopril. Therefore, we suggest that further study is warranted as to the potential for Captopril as additional option in the treatment for CCD. We present this case report with the informed consent of the patient's guardian.

## Case presentation

An 11 months 22 days old partially-breastfed female baby was admitted with the complaints of the passage of loose stool since birth, occasional vomiting, and failure to thrive. The stool was watery in nature; frequency was 8-10 times per day, and was not associated with blood or mucous. The parents were healthy but their marriage was consanguineous. Antenatal and birth history included polyhydramniosis and premature delivery at 35 weeks of gestation. The baby's birthweight was 2.1 kg. During her neonatal period, she was admitted to another hospital due to sepsis and hyperbillirubinaemia. Moreover, she had several episodes of hospitalization for the management of persistent diarrhoea before coming to our hospital. There was no history of cyanosis or congenital heart disease. On admission, the patient appeared toxic, had reduced activity, and signs of some dehydration [assessed by Dhaka Method (1) recommended by WHO]. Her weight on admission was 3.8 kg, and length was 61 cm. Pulse rate was 118/minute with BP 90/50 mm of Hg, temperature 36.8 ^o^C, respiration rate 48/minute, oxygen saturation (SpO_2_) 99% without applied oxygen on admission. On examination, no chest indrawing was observed, lungs were clear on auscultation, abdomen was soft, mildly distended but bowel sounds were present. She also showed signs of motor delay (neck control was not yet established).

Initially, the patient was managed as a case of chronic diarrhoea with some dehydration and severe acute malnutrition. We also suspected that her reduced activity might be due to electrolyte imbalance or sepsis. Hence, the following investigations were done: complete blood count (CBC), serum electrolyte and creatinine, blood and stool for culture and sensitivity (C/S), routine microscopic examination (RME) of stool and urine, and chest x-ray. The test report for electrolytes showed hypochloraemic hypokalaemia with alkalosis (Table 1 and 2) which, thereafter, became persistent. Based on these findings, our differential diagnosis now included gastric outlet obstruction, cystic fibrosis, Bartter Syndrome, and congenital chloride diarrhoea (CCD). Subsequent investigations showed normal chloride level in sweat, normal routine microscopy of stool, urinary Na^+^ was 16 mmol/L (reference value 54-150), K^+^ 24.52 mmol/L (reference value 20-80), and Cl^-^ 12 mmol/L (reference value 110-250). Tissue transglutaminase immunoglobulin (TtG-IgA) and IgG antibodies were negative, with plasma total protein 86.47 g/L (reference value 64.0-82.0), albumin 48.97 g/L (reference value 34.0-50.0), globulin 37.5 g/L (reference value 23.0-35.0), albumin and globulin ratio 1.31 (reference value 1.10-1.80). Chloride level in stool was 126.6 mmol/L (normal range 6-17 mmol/L), potassium 30.9 mmol/L, and sodium 82.9 mmol/L (normal range 50-60 mmol/L). The diagnosis of CCD was made on the basis of the results of faecal chloride content of >90 mmol/L and faecal chloride more than the sum of faecal Na and K contents and exclusion of other suspected diseases. Genetic testing could not be done due to its unavailability in Bangladesh. Urine and stool culture showed no growth of pathogens. We started oral NaCl and KCl supplementation and commercially-available oral formulation of Omeprazole. Other recommended treatment (with oral butyrate) was not applied as it was not available in Bangladesh. After 15 days of hospitalization, the patient developed fever and respiratory distress. The respiration rate was 64/minute, she was tachycardic (175 beats per minute), and the chest x-ray was suggestive of pulmonary infiltrates. So, we suspected that this was most likely due to a hospital-acquired infection and, initially, the patient was treated with injection Ceftazidime and injection Amikacin. However, no improvement was seen even after 5 days of treatment and, subsequently, the antibiotic was changed to injection Imipenem instead of ongoing medication. In the meantime, the blood C/S report was available, and it showed *Acinetobacter* species sensitive to Polymyxin B. Therefore, we started injection Polymyxin B. At that time, the patient also developed a changing murmur in addition to fever and anaemia, which raised the possibility of infective endocarditis. We, therefore, arranged an echocardiogram that showed mild coarctation of aorta, mild mitral regurgitation Grade 1, and mild pulmonary hypertension with normal ejection fraction (EF) of 70%. We added Captopril to protect heart failure. After adding Captopril, we found, to our surprise, that the stool output significantly reduced, and serum potassium increased. In response to this observation, we reduced the dose of supplemental KCL and NaCL and continued the treatment with Captopril. This decision was expedited by the fact that the overall condition of the patient was improving. The stool output and consistency was also improving. The patient attended regular follow-up with us, and the electrolyte profile had been normal in the next 6 months. In the first and second follow-up visit, we used Captopril with KCl (Table 2), and it showed that the diarrhoea was resolved and serum chloride and total carbon dioxide were also normal. The mother of the patient did not provide Captopril in the preceding one week prior to the third visit according to the advice of the local cardiologist, and the patient again developed alkalosis and hypokalaemia (Table 2). The most recent echocardiogram showed trivial MR, good biventricular function: left ventricular ejection fraction (LVEF) 69%, FS 34% left ventricular internal dimension in systole (LVIDs) 15 mm, left ventricular internal dimension in diastole (LVIDd) 24 mm trivial TR were noticed, along with peak pressure gradient (PPG) 10 mmHg, intact interatrial septum (IAS), and interventricular septum (IVS). No PDA or coarctation was seen. No intra-cardiac mass or vegetation was seen. Chamber dimensions and wall motion were normal. All valves were normal. No pericardial effusion was seen; biventricular function was good and, finally, the cardiac anatomy showed normal findings. The patient was gradually gaining weight from 3.8 kg to 7.1 kg in 7 months, and control over her neck was then completely normal. Her blood pressure was normal throughout the follow-up period.

**Table 1.** Test results for electrolytes in urine and stool

Random urine electrolytes				Random stool electrolytes			
Sodium	Chloride	Potassium	Calcium/Creatinine ratio	Magnesium	Sodium	Chloride	Potassium
15-20 mEq/L	15-20 mEq/L	15-20 mEq/L	<0.6	0.6-0.9 mmol/L	50-60 mmol/L	6-17 mEq/L	NA
16	12	18.8	Not done	Not done	82.9	126.6	30.2

NA=Not available

## DISCUSSION

Congenital chloride diarrhoea (CCD) is a rare hereditary disease with a prenatal onset, secondary to a deficit in intestinal chloride transport. It is more common in Finland, Poland, and the Middle East than in rest of the world. Diagnosis at neonatal period is often delayed in low-incidence regions, like Bangladesh and due to the lack of knowledge of and experience with the condition. This is the first reported case of CCD with positive effect of Captopril in the world.

**Table 2.** Test results for serum electrolytes

Parameter	Sodium	Chloride	Potassium	TCO**_2_**	Glucose	Creatinine	Diarrhoea
Normal value	135-45 mmol/L	97-106 mmol/L	3.5-5.3 mmol/L	18.0-24.0 mmol/L	3.3-5.6 mmol/L	18-35 μmol/L	Status
First test (after 5 days of KCL supplementation with ORS/occasional IV fluid)	137	82.45	2.52	31.79	5.5	23.4	Continued
Second test (after 3 days of cessation of KCL but ORS continued)	130.2	74.3	2.94	41.6	5.8	24.2	Continued
Third test (ORS continued)	134.95	68.73	2.87	50.18	5.2	33.3	Continued
Fourth test [after correction with IV (No ORS)]	140.77	79.13	3.96	43	4.6	45	Continued
Fifth test (Syp. KCL and Nacl) (with ORS and I/V)	137.7	104.4	4.99	26.0	5.2	15.0	Continued
After adding Captopril (with Syp. KCL and NaCl and ORS)	137.47	105.94	5.65	21.32	4.8	15.86	Reduced
One day after initiation of Captopril (ORS but no KCL or NaCl)	135.3	95.4	3.87	27.1	5.2	18.5	Resolved
After 7 days of last report (No KCL or NaCl)	137.09	92.34	3.41	30.98	4.8	15	Resolved
First follow-up after discharge (Captopril with ½ tsf of KCL twice a day)	136.6	91.91	3.14	26.7	4.6	24.5	Resolved
Secnd follow-up (Captopril with 3/4 tsf twice a day of KCL)	135.2	76.5	3.44	28.4	5.8	28.4	Resolved
Third follow-up (no Captopril but 1 tsf KCL and NaCL with Omeprazole) (after 6 months from the beginning)	138	97.7	3.33	33.4	4.8	29	Diarrhoea relapsed
Fourth follow-up (with Captopril and 1 tsf of KCL but no Omeprazole)) (after 7 months from the beginning)	135.11	100.52	3.56	25.43	5.5	18.8	Resolved with weight gain

IV=Intravenous fluid

TCO_2_=Total carbon dioxide

tsf=Teaspoonful

We have discussed a case of CCD, which was well-managed by Captopril, an angiotensin-converting enzyme-inhibitor drug. The first striking presentation of this female baby was hypochloraemic-hypokalaemic alkalosis with chronic diarrhoea since birth. The electrolyte imbalance was difficult to correct. The patient showed signs of some dehydration but it could not be corrected by oral replacement and required intravenous fluid. Unfortunately, the patient developed a hospital-acquired bacteraemia with *Acinetobacter* and was clinically diagnosed as a case of infective endocarditis and heart failure. After starting Captopril, we found that the watery portion of diarrhoeal stool significantly reduced. The stool output also reduced from an average 500 mL watery stool/day to 50 mL semi-solid stool/day. Supplementation of KCL and NaCl was not required.

Captopril prevents the conversion of angiotensin I to angiotensin II by inhibition of ACE, a peptidyl dipeptide carboxy hydrolase. Angiotensin II also stimulates aldosterone secretion from the adrenal cortex, thereby contributing to sodium and fluid retention. The reduction of angiotensin II leads to decreased aldosterone secretion, and, as a result, small increases in serum potassium may occur, along with sodium and fluid loss. On this basis, Captopril may have a role in reducing watery portion of diarrhoeal stool in CCD and should be further studied. CCD is the best-described congenital defect of large intestinal transport and characterized by watery diarrhoea with high levels of chloride in stool, which exceed the sum of Na and K contents in stool. The bicarbonate (HCO_3_) level and pH are also low in stool. CCD was first described in 1945 (2) and was due to a defect in anion exchange, leading to high chloride concentrations in stool. Theoretically, this may occur due to either failure of chloride absorption or to active secretion of chloride into the intestine. The alkalosis probably develops partly through an associated increase in H^+^ excretion and partly through an absence of HCO_3_ secretion in the ileum and proximal colon (3). The metabolic alkalosis can be due to either chloride-responsive alkalosis (urinary chloride <20 mEq/L) or chloride-resistant alkalosis (urinary chloride >20 mEq/L). We analyzed the urine sample of the patient for electrolyte concentrations to determine the mechanism at work and found that urinary chloride was 12 mmol/L (<20 mmol/L) and, hence, suggestive of a chloride-responsive alkalosis (Table 1). The causes of chloride-responsive alkalosis include loss of gastric secretions, such as vomiting and nasogastric (NG) suction; loss of colonic secretions, such as congenital chloride diarrhoea, villous adenoma, diuretics-use and cystic fibrosis (4). In chloride-responsive alkalosis with volume depletion, the alkalosis should be treated with an intravenous infusion of isotonic sodium chloride solution.

Furthermore, as this type of alkalosis is usually associated with hypokalaemia, it is also important to give potassium chloride supplements to correct this. Therefore, we corrected the alkalosis and hypokalaemia with normal saline, 5% dextrose in acqua (DA), and injection 20 mmol/L Potassium Chloride (Table 2). Biochemical abnormalities in CCD are similar to those in Pseudo-Bartter Syndrome, apart from urinary chloride content. This is low in untreated CCD but high in all forms of Bartter Syndrome. In cases of metabolic alkalosis, it is important to exclude Bartter Syndrome and cystic fibrosis. Infants with cystic fibrosis can also present with episodes of hyponatraemic, hypochloraemic dehydration with metabolic alkalosis, which are also biochemical hallmarks of the Pseudo-Bartter Syndrome (4). The chloride level in sweat was normal in our patient but is important to note that increased concentrations of chloride in sweat were reported in 12% of CCD patients (5).

Bartter Syndrome and Gitelman's Syndrome were less likely in our patient as these conditions are not typically associated with diarrhoea. The causes of Pseudo-Bartter Syndrome, such as chronic diuretics-use, chronic administration of a chloride-deficient diet, cyclic vomiting, and abuse of laxatives, were absent in our patient. To exclude Pseudo-Bartter Syndrome, it is important that the urine sample be collected appropriately by using a urine-bag. Other causes of congenital watery diarrhoea, such as congenital glucose-galactose malabsorption and congenital sodium diarrhoea result in metabolic acidosis instead of metabolic alkalosis. An increase in stool volume is mostly accompanied with respiratory infections (6). We have also excluded coeliac disease by negative tissue transglutaminase antibodies.

One of the late manifestations of CCD is growth retardation (7). However, other factors could have contributed to stunted growth in this patient; for example, repeated admission which interferes with wellbeing and appetite. Urinary tract infections were frequent in our patient but rare with our present mode of treatment. The possible explanation is that the defective absorption of chloride results in osmotic diarrhoea. Excessive fluid losses will subsequently lead to hypovolaemia and intravascular volume contraction. Hypovolaemia will activate the renin-angiotensin-aldosterone system, leading to high renin and secondary hyperaldosteronism which causes juxtaglomerular hyperplasia. In spite of the high levels of aldosterone, normovolaemia is difficult to maintain due to the constant loss of both water and salt through the intestine. Due to the constant state of hypovolaemia, hypertension is not seen in spite of the high angiotensin activity that leads to chronic vasoconstriction. On the other hand, Nephrocalcinosis occurs as a result of metabolic alkalosis, leading to alkaline urine which predisposes the deposition of calcium phosphate and oxalate salts and appearance of calcification in the distal convoluted tubules. Alkalinity of urine and renal calcifications are the likely factors responsible for the increased susceptibility to urinary tract infection (8). Besides this, the bacteria enter in the urinary tract by the ascending route through the urethra—possibly coming from faecal material. The renin-angiotensin-aldosterone system was partly blocked by Captopril and also improved the diarrhoeal condition. In the long run, incidence of renal impairment is high among the CCD patient, and, sometimes, it is difficult to prevent it with proper salt and fluid substitution therapy. The main focus of treatment for CCD is: (i) lifelong salt and potassium chloride substitution, (ii) early treatment for acute dehydration and hypokalaemia during gastroenteritis or other co-infections, and (iii) early recognition of and treatment for other complications of the disease, such as intestinal inflammation and renal impairment.

Historically, many therapeutic attempts were tried to treat CCD but, unfortunately, most had been found to be ineffective in reducing the severity of the disease and its long-term complications. Moreover, replacement therapy with oral NaCl and KCl substitution has been shown to ensure normal growth and development in children and partially prevent complications but it does not reduce the diarrhoea. Over-substitution must be avoided because it may even increase diarrhoea by an osmotic mechanism (9). Regular ORS and Omeprazole can significantly reduce the amount of water loss in diarrhoea but it does not completely prevent this. The patient developed iron-deficiency anaemia, which might be due to reduced iron absorption that was corrected by oral iron supplementation. Butyrate and cholestyramine can be used as diarrhoea-reducing therapies but these agents are unable to completely reduce the watery portion of stool (10,11).

We found a significant reduction in the watery portion of stool when we started Captopril in our patient, and diarrhoea was almost resolved over a longer period of treatment. The use of Captopril may help in reducing the recommended dose of potassium chloride supplementation by preventing the conversion of angiotensin I to angiotensin II described earlier in this paper. Acute exacerbations of electrolyte imbalance may also be labile and disturbed even by slight infections. The patient used to live with diarrhoea with an adequate social adjustment.

### Conclusions

The diagnosis of CCD by clinicians, even in resource-limited settings, may be simple, although it is potentially a fatal or disabling disease if remains undiagnosed and untreated. History of polyhydramnios, watery diarrhoea, failure to thrive, poor growth, metabolic alkalosis, hypokalaemia, or hypochloraemia may be an alarming set of findings for the early diagnosis that can be strengthened by faecal measurement of chloride content. Captopril may be the choice of drug in managing children with CCD, which may also help reduce the need for recommended dose of potassium and sodium supplementation in CCD.

## ACKNOWLEDGEMENTS

We are grateful to Dr. Mustofa Doha, Dr. Nasir Uddin, and Dr. Kazi Nazmus Saqeeb for their help in preparing the manuscript. We thank all clinicians and nurses of Dhaka Hospital of icddr,b, who were involved in taking care of the patient.

**Conflict of interest**: Authors declare no conflicts of interest and financial relationships relevant to this article.

## References

[1] Alam NH, Ashraf H (2003). Treatment of infectious diarrhea in children. Paediatr Drugs.

[2] Darrow DC (1945). Congenital alkalosis with diarrhoea. J Pediatr.

[3] Hochman HI, Feins NR, Rubin R, Gould J (1976). Chloride losing diarrhoea and metabolic alkalosis in an infant with cystic fibrosis. Arch Dis Child.

[4] Gennari FJ, Weise WJ (2008). Acid-base disturbances in gastrointestinal disease. Clin J Am Soc Nephrol.

[5] Özbay Hoşnut F, KaradağÖncel E, Öncel MY, Özcay F (2010). A Turkish case of congenital chloride diarrhea with SLC26A3 gene (c.2025_2026insATC) mutation: diagnostic pitfalls. Turk J Gastroenterol.

[6] Hihnala S, Höglund P, Lammi L, Kokkonen J, Ormälä T, Holmberg C (2006). Long-term clinical outcome in patients with congenital chloride diarrhea. J Pediatr Gastroenterol Nutr.

[7] Holmberg C, Perheentupa J, Launiala K, Hallman N (1977). Congenital chloride Diarrhoea. Clinical analysis of 21 Finnish patients. Arch Dis Child.

[8] Al-Hamad NM, Al-Eisa AA (2004). Renal abnormalities in congenital chloride diarrhea. Saudi Med J.

[9] Holmberg C, Perheentupa J, Launiala K, Hallman N (1977). Congenital chloride diarrhea: clinical analysis of 21 Finnish patients. Arch Dis Childhood.

[10] Höglund P, Holmberg C, Sherman P, Kere J (2001). Distinct outcomes of chloride diarrhea in two siblings with identical genetic background of the disease: implications for early diagnosis and treatment. Gut.

[11] Bieberdorf FA, Gorden P, Fordtran JS (1972). Pathogenesis of congenital alkalosis with diarrhea. Implications for the physiology of normal ileal electrolyte absorption and secretion. J Clin Invest.

